# Student's Pocket Medical Lexicon

**Published:** 1888-12

**Authors:** 


*Student's Pocket Medical Lexicon.* By ELIAS LONGLEY. A New Edition. Edinburgh: YOUNG J. PENTLAND. 1888.

Although this book bears the imprint of the well-known Edinburgh publisher, its appearance is so unmistakable, that in order to be assured of its trans-Atlantic origin, we wanted neither the author's address, which is given in the preface, nor the confirmation of its "American phonetic alphabet," by which the pronunciation is "plainly (!) represented." This alphabet contains so many terrifying Russian-like characters that we made no effort to master its detail, and we passed at once to an examination of the work itself. It contains no etymology; but with simple definitions, such as that which explains the neck to be "the part between the head and thorax," has such appalling looking words as "ecphyma," "emphyma," "inappetency," "pladarosis," "suggillation," "ulorrhagia," "vagitis." Nephrotomy is given, but neither nephrectomy nor nephorraphy, nor the analogous terms in reference to other organs; and in a book dated 1888 we expected to see definitions of leucomaines and ptomaines. Invagination is said to be "an operation for hernia by intro-susception;" typhoid fever is described as "a fever differing from the typhus only in the depressed condition of the intestines;" gastrotomy is "an incision for removing a fœtus or tumor;" and symphysotomy, a discredited operation, is vaguely described as "the operation of enlarging the diameter of the pelvis, to facilitate parturition." These are samples of the extraordinary things we have seen in this work.

In an American book which is produced on the phonetic system, it is hazardous to say that anything is a misprint; but "lasceration" (under Fractura), "galactirrhœa," "ovaduct" (p. 9), and "aqua ammonæ," probably are such.

A list of abbreviations used in prescriptions is given. All these, intended for the guidance of the patient, should be abolished. Directions should be written in full in the language of the country. Much misconception, leading to serious results, might thereby be avoided. In order to be convinced of this, there is no need to believe the apocryphal story of the druggist, who translated *pro re nata*, "for the thing just born," very much to the risk of the child, and very little for the benefit of the mother for whom the medicine was intended. There is a lengthy list of "Poisons and their Antidotes." The list is not merely a statement of antidotes in the chemical sense, but purports to give directions for treatment. Strange to say, there is no mention of the hypodermic use of apomorphia for producing vomiting.

Altogether this book leaves much to be desired, and cannot be recommended to the medical student, or anybody else.

